# Novel insights into cuproptosis inducers and inhibitors

**DOI:** 10.3389/fmolb.2024.1477971

**Published:** 2024-11-26

**Authors:** Ligang Zhang, Ruiting Deng, Lian Liu, Hongli Du, Dongsheng Tang

**Affiliations:** ^1^ Gene Editing Technology Center of Guangdong Province, School of Medicine, Foshan University, Foshan, China; ^2^ School of Biology and Biological Engineering, South China University of Technology, Guangzhou, China; ^3^ Beijing Mercer United International Education Consulting Co., Ltd., Guangzhou, China

**Keywords:** copper (Cu), cuproptosis, inducers and inhibitors, small molecule compounds, transcription factors, non-coding RNAs (ncRNAs)

## Abstract

Cuproptosis is a new pattern of Cu-dependent cell death distinct from classic cell death pathways and characterized by aberrant lipoylated protein aggregation in TCA cycle, Fe-S cluster protein loss, HSP70 elevation, proteotoxic and oxidative stress aggravation. Previous studies on Cu homeostasis and Cu-induced cell death provide a great basis for the discovery of cuproptosis. It has gradually gathered enormous research interests and large progress has been achieved in revealing the metabolic pathways and key targets of cuproptosis, due to its role in mediating some genetic, neurodegenerative, cardiovascular and tumoral diseases. In terms of the key targets in cuproptosis metabolic pathways, they can be categorized into three types: oxidative stress, mitochondrial respiration, ubiquitin-proteasome system. And strategies for developing cuproptosis inducers and inhibitors involved in these targets have been continuously improved. Briefly, based on the essential cuproptosis targets and metabolic pathways, this paper classifies some relevant inducers and inhibitors including small molecule compounds, transcription factors and ncRNAs with the overview of principle, scientific and medical application, in order to provide reference for the cuproptosis study and target therapy in the future.

## 1 Introduction

**FIGURE 1:**
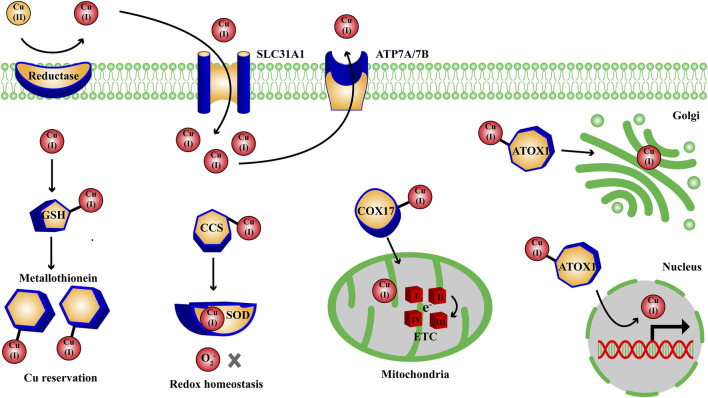
Overview on the intracellular metabolic pathways of Cu homeostasis. Extracellularly, Cu(II) are reduced to Cu(I) by metal reductase; intracellular Cu(I) are transported to nucleus, Golgi apparatus, SOD, mitochondria with the assistance of ATOX1, CCS, COX17 to participate in the metabolic process of gene regulation, protein folding, redox homeostasis and mitochondrial respiration through activating the target enzymes. ATOX1, antioxidant-1; CCS, Cu chaperone for superoxide dismutase; COX, cytochrome c oxidase; ETC, electron transport chain complexes; GSH, glutathione; SLC31A1, solute carrier family 31 member 1; SOD, superoxide dismutase.

Copper (Cu) is an indispensable co-factor of various crucial metabolic enzymes in a great deal of physiological processes, they will flow into metabolic disorders with aberrant Cu homeostasis (Xie et al., 2023). Thus the Cu level in human body must be maintained within a balanced range to ensure the normal cellular activities. Shellfish and organ meats tend to be the most abundant food sources for human absorbing Cu, and the intake of Cu mainly occurs in small intestine epitheliums, which should be recommended 0.8–2.4 mg/day for adult to maintain Cu homeostasis (Bost et al., 2016; Xie et al., 2023). The intracellular Cu level is regulated by a complex network of cuproenzymes, membrane transporters and Cu chaperones, which together coordinate the transport, reservation and intracellular metabolism of Cu, thereby keeping intracellular Cu level within a balanced range to prevent the damages of Cu overload and deficiency. With the assistance of specific Cu chaperones, intracellular Cu(I) are transported to the specific metabolic processes in cytoplasm and nucleus through activating the target enzymes: antioxidant-1 (ATOX1) carries Cu(I) to nucleus (gene regulation) and Golgi apparatus (protein folding), copper chaperone for superoxide dismutase (CCS) carries Cu(I) to superoxide dismutase (SOD) (redox homeostasis), and cytochrome c oxidase 17 (COX17) carries Cu(I) to mitochondria (mitochondrial respiration) (Figure 1) (Chen et al., 2022).

**FIGURE 2:**
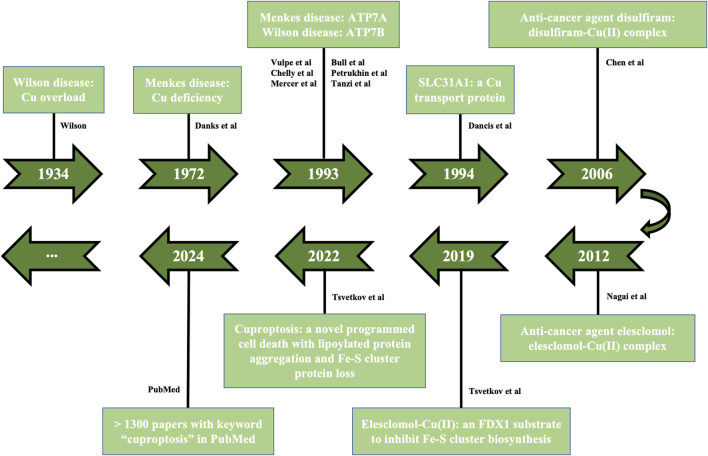
Tendency of cuproptosis development. The historical events contributing to studying Cu homeostasis and cuproptosis are displayed in the timeline from 1934 to 2024, involving Cu-mediated diseases (Wilson disease, Menkes disease), membrane transporters (ATP7A/7B, SLC31A1), anti-cancer agents of Cu complexes (disulfiram, elesclomol), action mode between elesclomol-Cu(II) and FDX1, the discovery of cuproptosis. FDX1, ferredoxin 1; SLC31A1, solute carrier family 31 member 1.

Cu-dependent cell death found in 2022 is a novel non-apoptotic programmed cell death, which accelerates the development of field of Cu homeostasis imbalance. There are few known mammalian lipoylated proteins and most of them are concentrated in tricarboxylic acid (TCA) cycle, while cells with active mitochondrial respiration show elevated lipoylated proteins (Tsvetkov et al., 2022; Chen et al., 2022; Xie et al., 2023). Excess Cu ions aggravate the insoluble aggregation of lipoylated proteins, reduce Fe-S cluster protein, cause proteotoxic stress, elevate heat shock protein 70 (HSP70) and reactive oxygen species (ROS), ultimately leading to cell death effects, named cuproptosis. The discovery of cuproptosis is inseparable from the progress of previous studies on Cu homeostasis and Cu-induced cell death, involving Cu-mediated diseases [Wilson disease (Wilson, 1934), Menkes disease (Danks et al., 1972)], membrane transporters [ATP7A (Vulpe et al., 1993; Chelly et al., 1993; Mercer et al., 1993), ATP7B (Bull et al., 1993; Petrukhin et al., 1993; Tanzi et al., 1993), solute carrier family 31 member 1 (SLC31A1) (Dancis et al., 1994)], anti-cancer agents of Cu complexes [disulfiram (Chen et al., 2006), elesclomol (Nagai et al., 2012)], action mode between elesclomol-Cu(II) and ferredoxin 1 (FDX1) (Tsvetkov et al., 2019) (Figure 2).

Based on the characteristic changes, cuproptosis is a new form of Cu-dependent cell death distinct from traditional cell death including apoptosis, autophagy, ferroptosis, pyroptosis, necrosis, and Cu homeostasis imbalance will lead to some adverse health effects, involving genetic, neurodegenerative, cardiovascular, tumoral diseases (Tsvetkov et al., 2022; Chen et al., 2022). For genetic diseases: Menkes disease and Wilson disease, caused by mutations in ATP7A/7B gene, generalize severe organ Cu deficiency or overload. For neurodegenerative diseases: in Alzheimer’s disease, Cu ions may interplay with the key causative factors like amyloid-β peptides and tau protein; in amyotrophic lateral sclerosis, Cu deficiency is reported to promote the aberrant hydrophobicity of pathogenic mutant SOD1; in Huntington’s disease, Cu overload occurs in the striatum of patients and mouse models. For cardiovascular diseases: excess Cu in relation to atherosclerosis while Cu deficiency in relation to cardiac hypertrophy. For tumoral diseases: Cu is involved in the malignant progression of cancer by facilitating cell proliferation, angiogenesis and metastasis. Hence, comprehensive exploration of potential mechanism of Cu homeostasis in disease development, and in-depth study of Cu homeostasis-associated regulatory pathways under different pathological areas have grand medical value and translational significance.

With the emergency of cuproptosis, scientific communities pay more attention to Cu-induced cell death owing to its enormous potential in disease target therapy. Nowadays, a growing body of studies have revealed the target molecules and metabolic pathways in response to Cu cell death effects. Meanwhile, inducers and inhibitors of cuproptosis have been widely applied in scientific and medical areas. However, the targets and metabolic pathways of Cu-dependent cell death are not totally explained, which limits the development and application of cuproptosis inducers and inhibitors. In this study, we intend to briefly summarize the essential targets and metabolic pathways of cuproptosis, then categorize commonly used and newly discovered inducers and inhibitors with the overview of principle, scientific and medical application, and discuss their future development tendency, hoping to increase the understanding of the inducers and inhibitors in research field and disease target therapy based on cuproptosis.

## 2 The key targets in cuproptosis metabolic pathways

**FIGURE 3:**
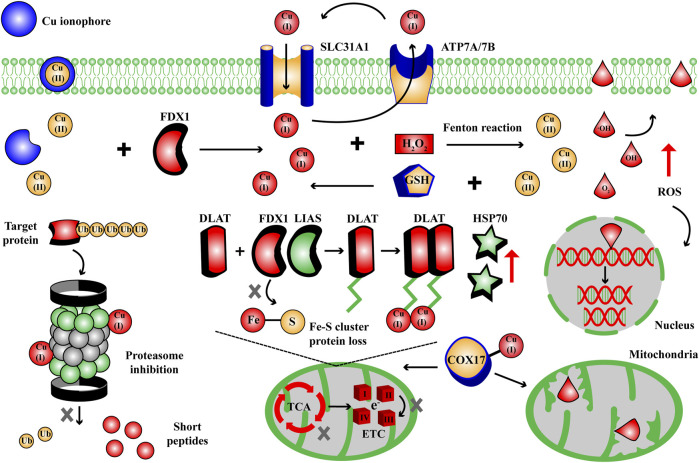
The targets and metabolic pathways of cuproptosis mechanism. During cuproptosis, excess Cu ions can cause Fenton reaction to generate ROS, disrupt lipoylated pathway in TCA cycle and Fe-S cluster protein, trigger UPS inhibition, in response to Cu cell death effects. COX, cytochrome c oxidase; DLAT, dihydrolipoamide S-acetyltransferase; ETC, electron transport chain complexes; FDX1, ferredoxin 1; GSH, glutathione; HSP70, heat shock protein 70; LIAS, lipoyl synthase; ROS, reactive oxygen species; SLC31A1, solute carrier family 31 member 1; TCA, tricarboxylic acid cycle; Ub, ubiquitin.

The metabolic homeostasis of metal elements play a key role in internal environment and normal metabolism. The overload or deficiency of metal elements will disrupt and impair metabolic homeostasis, like iron and ferroptosis (Dixon et al., 2012). Over the past decade, multiple types of ferroptosis inducers and inhibitors have been employed in practice in terms of the targets and metabolic pathways involved in Fe-dependent cell death (Du and Guo, 2022). Similarly, the strategies of cuproptosis inducers and inhibitors have been continuously improved based on the key targets in cuproptosis metabolic pathways (Figure 3): oxidative stress, mitochondrial respiration, ubiquitin-proteasome system (UPS).

### 2.1 Oxidative stress: excess Cu ions cause Fenton reaction to generate ROS

Tsvetkov et al. found that elesclomol elevated tenfold intracellular Cu ion level to trigger cell death effects 24 h later (Tsvetkov et al., 2022). Cu is one of the transition metals and accumulation of Cu ions can generate massive ROS via Fenton reaction: Cu(I) and H_2_O_2_ generate Cu(II), ·OH, OH^−^ and O_2_; glutathione (GSH) reduces Cu(II) to enhance Cu(I)/H_2_O_2_ reaction, which causes lipid peroxidation, disrupts cytomembrane integrity, triggers DNA damage and mitochondrial dysfunction (Jomova et al., 2022; Fu et al., 2021; Li et al., 2022). Thus excess intracellular Cu ions and ROS are one of the biochemical features in Cu-dependent cell death. How to elevate or reduce the content of intracellular Cu ions and ROS will be the key targets to induce or inhibit cuproptosis process.

### 2.2 Mitochondrial respiration: excess Cu ions induce cell death effects by targeting lipoylated pathway in TCA cycle and Fe-S cluster protein

According to the discovery of Tsvetkov et al., 10 genes have been identified as cuproptosis-related genes. Among them, FDX1 and lipoylated proteins including lipoic acid pathway lipoacyltransferase 1 (LIPT1), lipoyl synthase (LIAS), dihydroacylamide dehydrogenase (DLD) and lipoylated targets dihydrolipoamide S-acetyltransferase (DLAT), pyruvate dehydrogenase E1 subunit α 1 (PDHA1), pyruvate dehydrogenase E1 subunit β (PDHB) act as positive regulatory factors, while glutaminase (GLS), cyclin-dependent kinase inhibitor 2A (CDKN2A), metal regulatory transcription factor 1 (MTF1) generate negative regulatory effects (Tsvetkov et al., 2022). The vital characteristic and target of cuproptosis is lipoylated pathway, a conserved post-translational lysine modification which is important for the key enzymes like DLAT regulating TCA cycle. DLAT is an essential component of PDH complex, which catalyzes the decarboxylation of pyruvate to acetyl-CoA in TCA cycle, and lipoylation of DLAT is required for its enzymatic function (Rowland et al., 2018). Excess Cu ions (Cu(I)) bind to the lipoylated DLAT, then the insoluble oligomerization of DLAT and the loss of Fe-S cluster protein result in proteotoxic stress and cell death, accompanied with HSP70 elevation. So FDX1 is the key regulator for cuproptosis development: firstly, FDX1 reduces excess intracellular Cu(II) to Cu(I); secondly, FDX1 and LIAS act as the upstream factors regulating the lipoylation of DLAT; thirdly, Fe-S cluster protein functions as the prosthetic groups of many enzymes essential for mitochondrial metabolism (e.g., LIAS and mitochondrial complex I/II) and FDX1 regulates its biosynthesis, but reducing excess Cu(II) disrupts the normal activity of FDX1 (Zulkifli et al., 2023; Sheftel et al., 2010; Cameron et al., 2011). Thus the discovery of cuproptosis reveals that the targets and pathways involved in Cu-dependent cell death are highly correlated with mitochondrial respiration proteins, showing increasing expect for drug discovery.

### 2.3 UPS: Cu ions inhibition

UPS is a key protein degradation pathway for regulating cell proliferation and apoptosis, which selectively connects ubiquitin to the redundant proteins and signals them for degradation, but UPS inhibition will trigger cytochrome c release to cytoplasm and activate the caspase cascade for apoptosis (Sidor-Kaczmarek et al., 2017). Studies have shown that Cu(II) complexes exhibit excellent inhibition of UPS via direct binding and redox reaction (Panebianco et al., 2023; Zhang and Burke, 2023). Interestingly, the rapid proliferation of tumor cells is more dependent on the metabolic support of UPS, so they will be more sensitive to metal complex-induced proteasome inhibition. Chen et al. found that disulfiram-Cu(II) complexes induced the apoptosis effects of breast cancer cells by inhibiting proteasome activity and increasing ubiquitin-protein interaction, while the study of Skrott et al. showed that disulfiram-Cu(II) complexes suppressed UPS and led to the cell death of breast cancer by blocking signaling upstream (Chen et al., 2006; Skrott et al., 2017). Therefore UPS inhibition is responsible for the another metabolic pathway involved in Cu-dependent cell death, which can not be neglected for the design strategy of cuproptosis inducers and inhibitors.

## 3 Cuproptosis inducers

**FIGURE 4:**
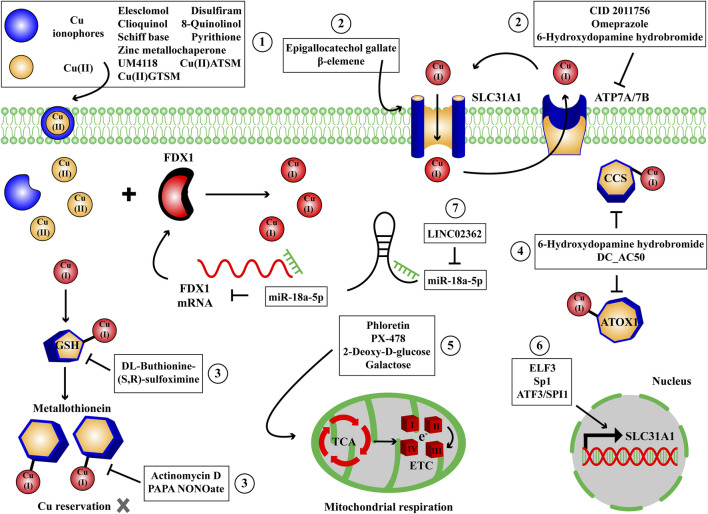
The small molecule compounds, transcription factors and ncRNAs to induce Cu-dependent cell death. We have collected some small molecule compounds targeting Cu ionophore ①, membrane transporter ②, reservation ③, chaperone ④, mitochondrial respiration and TCA cycle ⑤, transcription factors targeting membrane transporter ⑥ and ncRNAs ⑦ targeting FDX1 to induce Cu-dependent cell death. ATOX1, antioxidant-1; CCS, Cu chaperone for superoxide dismutase; ETC, electron transport chain complexes; FDX1, ferredoxin 1; GSH, glutathione; SLC31A1, solute carrier family 31 member 1; TCA, tricarboxylic acid cycle.

The proper cuproptosis inducers should cause intracellular Cu ions overload and lead to Cu-dependent cell death via the characteristic targets and metabolic pathways of cuproptosis mentioned above. With the discovery of cuproptosis, inducers of Cu-dependent cell death receive increasing concerns and novel inducers are constantly invented. Since cells can not suffer Cu overload, it suggests that the accumulation of Cu ions enables to selectively kill the morbid cells. Therefore cuproptosis inducers show promising application values both in scientific and medical areas. And choosing an appropriate inducer to induce Cu-dependent cell death can not divorce from the properties of inducers, targets, relevant studies and diseases. In this section, based on the characteristic targets and metabolic pathways of cuproptosis, the cuproptosis inducers we collect are categorized into small molecule compounds, transcription factors and non-coding RNAs (ncRNAs) (Figure 4; Table 1).

**TABLE 1:** Display of cuproptosis inducers.

Agents	Types	Targets	Medical value	Ref.
Small molecule compounds				
Elesclomol	Cu ionophore	FDX1	Cardiopathy, Menkes, cancer	Guthrie et al. (2020), O’Day et al. (2009), Monk et al. (2018)
Disulfiram	Cu ionophore	ALDH, NPL4	Alcohol dependence, cancer	Skrott et al. (2017), Viola-Rhenals et al. (2018), Huang et al. (2016)
Clioquinol	Cu ionophore	Proteasome	Bacteria, cancer, Alzheimer	Schimmer (2011), Katsuyama et al. (2021), Adlard et al. (2008)
8-Quinolinol	Cu ionophore	Proteasome	Bacteria, cancer	Zhai et al. (2010)
Schiff base	Cu ionophore	Proteasome	Microbia, oxidative stress, inflammation, cancer	Kumar et al. (2024)
Pyrithione	Cu ionophore	Proteasome	Microbia, cancer	Chen et al. (2017)
Zinc metallochaperone	Cu ionophore	P53 mutants	Cancer	Zaman et al. (2019)
Cu(II)ATSM Cu(II)GTSM	Cu ionophore	SOD, glycogen synthase kinase 3β	Amyotrophic lateral sclerosis, cancer, Alzheimer	Soon et al. (2011), Andres et al. (2020), Crouch et al. (2009)
UM4118	Cu ionophore	SF3B1	Cancer	Moison et al. (2024)
Epigallocatechol gallate	Cu membrane transporter	SLC31A1	Cancer	Chen et al. (2020)
β-elemene	Cu membrane transporter	SLC31A1	Cancer	Li et al. (2016)
CID 2011756	Cu membrane transporter	ATP7A/7B	Cancer	Janardhanan et al. (2022)
Omeprazole	Cu membrane transporter	ATP7A	Cancer	Matsui et al. (2015)
6-Hydroxydopamine hydrobromide	Cu membrane transporter/chaperone	ATP7A, ATOX1	Parkinson	Kondo et al. (2021)
Actinomycin D	Cu reservation	Metallothionein	Cancer	Steinebach and Wolterbeek (1994)
PAPA NONOate	Cu reservation	Metallothionein	Wound healing	Liu et al. (2000)
DL-Buthionine-(S,R)-sulfoximine	Cu reservation	GSH	Cancer	Steinebach and Wolterbeek (1994)
DC_AC50	Cu chaperone	ATOX1, CCS	Cancer	Wang et al. (2015)
Phloretin	Mitochondrial respiration and TCA cycle	GLUT1	Inflammation, cancer	Zhang et al. (2024)
PX-478	Mitochondrial respiration and TCA cycle	HIF-1α	Cancer	Li et al. (2024)
2-Deoxy-D-glucose	Mitochondrial respiration and TCA cycle	Hexokinase	Cancer	Yang et al. (2023)
Galactose	Mitochondrial respiration and TCA cycle	—	Cancer	Tsvetkov et al. (2022), Yang et al. (2023)
Transcription factors				
ELF3	Transcription factor	SLC31A1	Acute kidney injury	Qiu et al. (2024)
Sp1	Transcription factor	SLC31A1	Cancer, intervertebral disc degeneration	Song et al. (2008), Chen et al. (2024)
ATF3/SPI1	Transcription factor	SLC31A1	Cardiomyopathy	Huo et al. (2023)
NcRNAs				
LINC02362/miR-18a-5p	LncRNA/miRNA	FDX1	Cancer	Quan et al. (2023)

ALDH, aldehyde dehydrogenase; ATOX1, antioxidant-1; CCS, Cu chaperone for superoxide dismutase; FDX1, ferredoxin 1; GLUT1, glucose transporter 1; GSH, glutathione; HIF-1α, hypoxia inducible factor 1α; NPL4, nuclear protein localization 4; SLC31A1, solute carrier family 31 member 1; SOD, superoxide dismutase; TCA, tricarboxylic acid cycle.

### 3.1 Small molecule compounds to raise intracellular Cu content by targeting Cu ionophores, membrane transporters, reservation and chaperones

#### 3.1.1 Cu ionophores

As lipophilic compounds, Cu ionophores are reversible copper complexes that deliver Cu(II) into cells. In the study of Tsvetkov et al., researchers tested 1,448 compounds and screened against 489 cell lines from PRISM Repurposing Secondary dataset to verify drugs with growth inhibition similar to elesclomol (Tsvetkov et al., 2022). Hereby, Cu ionophores have received lots of concerns which enable to effectively raise intracellular Cu content and obtain favorable therapeutic outcomes through triggering Cu-dependent cell death. Elesclomol: the formal condensation of malonic acid carboxy groups and two molar equivalents of N-methylbenzenecarbothiohydrazide hydrino groups generate elesclomol; elesclomol-Cu(II) complex binds to the α2/α3 helix and β5 chain of FDX1, but does not bind to the parologous protein FDX2; 40 nM elesclomol-Cu(II) resulted in a tenfold increase in intracellular Cu level in 2 h and triggered ABC1 cell death after 24 h (Tsvetkov et al., 2022); elesclomol displays dramatic medical value by raising intracellular Cu content, such as improving serious cardiac pathology in cardiac SLC31A1 knockout mice, preventing harmful neurodegenerative changes in Menkes murine model (Guthrie et al., 2020) and killing cancer cells (O’Day et al., 2009; Monk et al., 2018). Disulfiram: DSF is a dithiocarbamate compound that treats alcohol dependence by targeting aldehyde dehydrogenase (ALDH), while acid condition reduces DSF to diethyldithiocarbamate (DTC) and invests the capacity to combine Cu(II) (Viola-Rhenals et al., 2018); DTC-Cu(II) complex induces lethal effects in ALDH^+^ and nuclear protein localization 4 positive (NPL4^+^) cancer cells combined with Cu overload (Skrott et al., 2017; Huang et al., 2016). Clioquinol: it is an oral anti-parasitic agent and recently displays preclinical efficacy in malignancy through inhibiting proteasome and directing Cu to proteasome and in Alzheimer through recovering intracellular Cu content (Schimmer, 2011; Katsuyama et al., 2021; Adlard et al., 2008). In addition to clioquinol-Cu, many Cu complexes have been exploited as proteasome inhibitors, e.g., 8-quinolinol-Cu (Zhai et al., 2010), schiff base-Cu (Kumar et al., 2024), pyrithione-Cu (Chen et al., 2017) and so on. Other Cu ionophores including P53 mutant reactivator zinc metallochaperone (Zaman et al., 2019), bis-thiosemicarbazone Cu(II) complexes Cu(II)ATSM/Cu(II)GTSM (Soon et al., 2011; Andres et al., 2020; Crouch et al., 2009) and C7-locked N-(quinoline-8-yl)benzenesulfonamide analogue UM4118 (Moison et al., 2024) are also undergoing preclinical studies of tumoral and neurodegenerative diseases.

#### 3.1.2 Cu membrane transporters

SLC31A1 and ATP7A/7B are classic membrane transporters that mediate Cu(I) endocytosis and expulsion, thus using small molecule compounds to regulate SLC31A1 and ATP7A/7B may be a valid strategy to control intracellular Cu content and cuproptosis. Epigallocatechol gallate, a green tea polyphenol (Chen et al., 2020), β-elemene, a curcuma wenyujin plant extract (Li et al., 2016), they can upregulate SLC31A1 expression; CID 2011756, a protein kinase D inhibitor (Janardhanan et al., 2022), omeprazole, a proton pump inhibitor (Matsui et al., 2015), 6-hydroxydopamine hydrobromide, a neurotoxin (Kondo et al., 2021), they can downregulate ATP7A/7B expression. These compounds are employed for cancer and Parkinson therapy. Notably, in 2023, Solier S et al. identified CD44 as a novel Cu membrane transporter that endocytosed Cu(II) into inflammatory macrophages, hereby CD44 would be another candidate for cuproptosis drug discovery based on Cu membrane transporter (Solier et al., 2023).

#### 3.1.3 Cu reservation

The intracellular Cu(I) is complexed by GSH and transferred to metallothionein, in which sulfur makes the binding to Cu(I) and the redox cycle regulates the binding and release. The cellular oxidants cause metals release and generation of metallothionein-disulfide, which can be reduced by GSH (Kang, 2006). Thus inhibition of GSH and metallothionein function can aggravate the release of Cu(I) to raise the cellular free Cu(I) in Cu stress, so as to induce Cu cytotoxicity. Actinomycin D, a DNA repair inhibitor, can decrease metallothionein expression (Steinebach and Wolterbeek, 1994); PAPA NONOate, a NO donor, can oxidize metallothionein-sulfur (Liu et al., 2000); DL-Buthionine-(S,R)-sulfoximine serves as an inhibitor of GSH synthesis (Steinebach and Wolterbeek, 1994). These compounds can be employed to disrupt the Cu reservation mediated by GSH-metallothionein so that dysfunction Cu homeostasis.

#### 3.1.4 Cu chaperones

ATOX1, CCS, COX17 carry intracellular Cu(I) to nucleus, Golgi apparatus, SOD, mitochondria, respectively and modulate normal metabolic activities, thereby inhibition of Cu chaperones will reduce Cu trafficking, another approach to aggravate Cu accumulation via small molecule compounds (Chen et al., 2022). Wang et al. reported a small molecule DC_AC50, inhibitor of ATOX1 and CCS, disrupted intracellular Cu transport and triggered oxidative stress in cancer cells (Wang et al., 2015). Thus DC_AC50 is also a potential cuproptosis inducer to mediate Cu-dependent cell death, but the specific COX17 inhibitor awaits further exploration.

### 3.2 Small molecule compounds to induce cuproptosis by targeting mitochondrial respiration and TCA cycle

Tsvetkov P et al. verified that Cu stress was more sensitive to the cells with active mitochondrial respiration and high levels of lipoylated TCA enzymes. Further, 10 genes have been identified as cuproptosis-related genes including positive regulatory factors FDX1, lipoylated proteins (LIPT1, LIAS, DLD), lipoylated targets (DLAT, PDHA1, PDHB) and negative regulatory factors GLS, CDKN2A, MTF1 (Tsvetkov et al., 2022). AT-rich interactive domain-containing protein 1A is a mutated tumor suppressor gene in hepatocellular carcinoma, and the data of Xing T et al. found that loss of this gene shifted the glucose metabolism of tumor cells from glycolysis to TCA cycle and oxidative phosphorylation (OXPHOS), which upregulated FDX1 and lipoylated proteins and exhibited greater sensitivity to Cu stress in tumor cells (Xing et al., 2023). The small molecule compounds that suppress glycolysis and favor OXPHOS may thus be promising tools for killing cells sensitive to cuproptosis. Phloretin (glucose transporter (GLUT) inhibitor) (Zhang et al., 2024), PX-478 (hypoxia inducible factor 1α (HIF-1α) inhibitor) (Li et al., 2024), 2-deoxy-D-glucose (hexokinase inhibitor) (Yang et al., 2023), galactose (Tsvetkov et al., 2022; Yang et al., 2023), these glycolysis inhibitors would have been taken into therapeutic consideration for cancer sensitive to cuproptosis through driving reprogramming of TCA cycle and OXPHOS.

### 3.3 Transcription factors to induce cuproptosis

Transcription factors regulate the expression of target genes through the binding to promoters, and the specific transcription factors in response to manipulating cuproptosis-related genes have been continuously identified. According to the chromatin immunoprecipitation and luciferase reporter analysis, ELF3 was found to directly bind to the promoter of SLC31A1 and promote its expression, which imbalanced Cu homeostasis and mitochondrial function to exacerbate acute kidney injury during cisplatin chemotherapy (Qiu et al., 2024). In small cell lung cancer cells, Sp1 could function as a positive regulator for SLC31A1 expression and the influence of Sp1 to intracellular Cu concentration was reliant on its zinc finger motif (Song et al., 2008). In intervertebral disc degeneration, oxidative stress promoted the Sp1-mediated SLC31A1 transcription, leading to increased TCA cycle-related protein aggregation and Cu-induced cytotoxicity (Chen et al., 2024). In cardiac dysfunction, ATF3 and SPI1 were validated to control the expression of SLC31A1 in cardiomyocytes, which exhibited features of cuproptosis (Huo et al., 2023). Thus these transcription factors we collect above are involved in either pathogenesis or therapeutic strategy based on regulating Cu membrane transporters. Mechanically, the investigation of cuproptosis-related transcription factors contributes to understanding the hallmark of Cu-dependent cell death and discovering appropriate drugs if they bore the capacity to modulate cuproptosis-related genes.

### 3.4 NcRNAs to induce cuproptosis

ncRNAs are a kind of functionl RNA molecules that are not translated into proteins. Regulatory ncRNAs play critical roles in post-transcriptional events regulating cuproptosis-related genes, which have attracted considerable interests and been intensively studied. LncRNAs, cirRNAs and miRNAs have been reported to modulate the expression of SLC31A1, ATP7A/7B, FDX1 and other cuproptosis-related genes, but the studies involved are mainly subject to reverse drug resistance before the discovery of cuproptosis and rarely referred to Cu-dependent cell death so far (Quan et al., 2023; Fu et al., 2022; Feng et al., 2018; Yu et al., 2020). The data of Quan et al. provided the evidence that the binding of LINC02362 to miR-18a-5p served as a molecular sponge to modulate FDX1, which bolstered the sensitivity of hepatocellular carcinoma to oxaliplatin via cuproptosis (Quan et al., 2023). Further research is essential to investigate comprehensive functions of ncRNAs to Cu homeostasis and cuproptosis, but it remains overwhelming difficulties to deliver ncRNAs to human body.

## 4 Cuproptosis inhibitors

**FIGURE 5:**
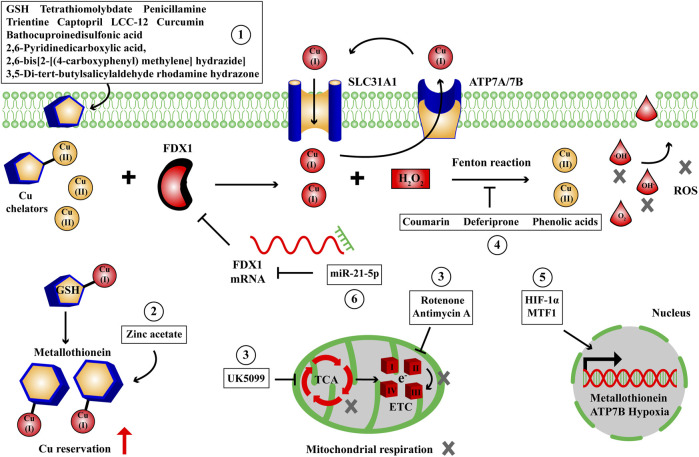
The small molecule compounds, transcription factors and ncRNAs to suppress Cu-dependent cell death. We have collected some small molecule compounds targeting Cu chelator ①, reservation ②, mitochondrial respiration and TCA cycle ③, oxidative stress ④, transcription factors targeting energy metabolism, membrane transporter, reservation ⑤ and ncRNA ⑥ targeting FDX1 to suppress Cu-dependent cell death. ETC, electron transport chain complexes; FDX1, ferredoxin 1; GSH, glutathione; HIF-1α, hypoxia inducible factor 1α; MTF1, metal regulatory transcription factor 1; ROS, reactive oxygen species; SLC31A1, solute carrier family 31 member 1; TCA, tricarboxylic acid cycle.

The eligible cuproptosis inhibitors should antagonize the cell death effects of Cu overload and alleviate the cellular damages induced by cuproptosis including oxidative stress, mitochondrial respiration dysfunction and UPS inhibition. Tsvetkov and colleagues verified that the cytotoxicity of Cu ionophore was eliminated by Cu chelator (tetrathiomolybdate) rather than other pathway inhibitors including Z-VAD-FMK, Boc-D-FMK (apoptosis), ferrostatin-1 (ferroptosis) and necrostatin-1 (necroptosis), whereas other metal chelators such as deferoxamine mesylate [high affinity for Fe(III)] and TPEN [high affinity for Zn(II)] had no effect on the killing capacity induced by Cu ionophore (Tsvetkov et al., 2022). Mechanically, the Cu cell death effects depended on mitochondrial respiration and TCA cycle, since treatment with mitochondrial antioxidants, fatty acids, mitochondrial function inhibitors induced distinct effects on Cu ionophore sensitivity; and furthermore treatment with electron transport chain complexes (ETC) inhibitors, mitochondrial pyruvate uptake inhibitors attenuated the cell death; treatment with mitochondrial uncoupler FCCP had no effect on Cu cytotoxicity (Tsvetkov et al., 2022). The scientific and medical communities have continuously developed available drugs for the diseases in Cu overload, and the discovery of cuproptosis significantly accelerates the progress of cuproptosis inhibitors. Based on the targets and metabolic pathways involved, like inducers, the cuproptosis inhibitors we collect can be similarly categorized into small molecule compounds, transcription factors and ncRNAs (Figure 5; Table 2).

**TABLE 2:** Display of cuproptosis inhibitors.

Agents	Types	Targets	Medical value	Ref.
Small molecule compounds				
GSH	Cu chelator	Cu ions	Oxidative stress	Tsvetkov et al. (2022)
Tetrathiomolybdate	Cu chelator	Cu ions	Wilson, cancer	Ishida et al. (2013), Pass et al. (2008), Henry et al. (2006), Kirk et al. (2024), Brewer et al. (2006)
Bathocuproinedisulfonic acid	Cu chelator	Cu ions	Cancer	Tsvetkov et al. (2022)
Trientine	Cu chelator	Cu ions	Wilson, cancer	Ala et al. (2015), Schilsky et al. (2022), Moriguchi et al. (2002)
Penicillamine	Cu chelator	Cu ions	Cardiovascular, Wilson, cancer	Schilsky et al. (2022), Mammoto et al. (2013), Wang D. et al. (2023)
2,6-Pyridinedicarboxylic acid, 2,6-bis[2-[(4-carboxyphenyl) methylene] hydrazide]	Cu chelator	Cu ions	Alzheimer	Singh et al. (2013)
3,5-Di-tert-butylsalicylaldehyde rhodamine hydrazone	Cu chelator	Cu ions	Alzheimer	Chauhan et al. (2022)
Captopril	Cu chelator	Cu ions	Hypertension, oxidative stress	Tamba and Torreggiani (2000)
LCC-12	Cu chelator	Cu ions	Inflammation	Solier et al. (2023)
Curcumin	Cu chelator	Cu ions	Cancer	Zhang et al. (2018)
Zinc acetate	Cu reservation	Metallothionein	Wilson	Shimizu et al. (2010), Munk et al. (2022)
Rotenone	Mitochondrial respiration and TCA cycle	ETC	Microbia	Tsvetkov et al. (2022), Kishore Kumar et al. (2017)
Antimycin A	Mitochondrial respiration and TCA cycle	ETC	Microbia	Tsvetkov et al. (2022), Lanju et al. (2014)
UK5099	Mitochondrial respiration and TCA cycle	Mitochondrial pyruvate uptake	Cancer	Tsvetkov et al. (2022), Wang et al. (2018)
Coumarin	Oxidative stress	Fenton reaction	Inflammation	Filipský et al. (2015)
Deferiprone	Oxidative stress	Fenton reaction	Inflammation	Timoshnikov et al. (2019)
Phenolic acids	Oxidative stress	Fenton reaction	Inflammation	Castañeda-Arriaga et al. (2021)
Transcription factors				
HIF-1α	Transcription factor	Hypoxia	Cancer	Huang et al. (2024)
MTF1	Transcription factor	ATP7B, metallothionein	Wilson	Stalke et al. (2020), Adams et al. (2015)
NcRNA				
MiR-21-5p	MiRNA	FDX1	Cancer	Xie et al. (2022)

ETC, electron transport chain complexes; FDX1, ferredoxin 1; GSH, glutathione; HIF-1α, hypoxia inducible factor 1α; MTF1, metal regulatory transcription factor 1; TCA, tricarboxylic acid cycle.

### 4.1 Small molecule compounds to reduce intracellular Cu content by targeting Cu chelators and reservation

#### 4.1.1 Cu chelators

In contrast to ionophores delivering Cu ions to cells, Cu chelators are another type of Cu complexes that reduce intracellular Cu content and perturbate cuproptosis pathways. In the study of Tsvetkov et al., two Cu chelators tetrathiomolybdate and bathocuproinedisulfonic acid are used to eliminate the cell death effects of Cu ions (Tsvetkov et al., 2022). Tetrathiomolybdate is an anti-Cu agent exerting anti-tumor effects in animal model and clinical trial (NCT00150995) by reducing tumor growth and angiogenesis (Ishida et al., 2013; Pass et al., 2008; Henry et al., 2006) and used for Wilson disease therapy in clinical trial (NCT00004339) by reducing Cu deposition in liver and brain (Kirk et al., 2024; Brewer et al., 2006). GSH is an endogenous intracellular Cu chelator for Cu metabolic balance, while various Cu chelators developed to date have been well-studied in animal model and clinical trial. Among them, the chelating effect of trientine can treat Wilson disease as well as inhibit the angiogenesis process of hepatocellular carcinoma through reducing the angiogenic factor production (Schilsky et al., 2022; Moriguchi et al., 2002); penicillamine has been shown to retard glioblastoma progression by decreasing lysyl oxidase enzymatic activity and angiogenic effect, apart from cardiovascular and Wilson diseases therapy (Schilsky et al., 2022; Mammoto et al., 2013; Wang D. et al., 2023); the synthesized 2,6-Pyridinedicarboxylic acid, 2,6-bis[2-[(4-carboxyphenyl) methylene] hydrazide] and 3,5-di-tert-butylsalicylaldehyde rhodamine hydrazone bore the capacity to counter Cu ions to rescue the deposits of amyloid-beta peptide in Alzheimer’s disease model of *Drosophila* (Singh et al., 2013; Chauhan et al., 2022); captopril, an angiotensin converting enzyme inhibitor for hypertension, is reported to protect against free radical by chelation to Cu (Tamba and Torreggiani, 2000); other Cu chelators include LCC-12, a derivative of metformin with anti-inflammatory effects and curcumin, a naturally occurring polyphenol with anti-tumor activity (Solier et al., 2023; Zhang et al., 2018). Therefore Cu chelators can be employed as the medical options of Cu homeostasis dysfunction through alleviating Cu overload and preliminarily applied to identify cuproptosis via rescuing the Cu cell death effects in Cu homeostasis studies.

#### 4.1.2 Cu reservation

Facilitating cellular metallothionein expression may reduce the release of Cu ions and prevent the Cu cell death effects. Zinc acetate is one such notable small molecule compound developed for the treatment of Wilson disease, which induces the expression of metallothionein in intestinal cells to block Cu absorption and reduce Cu deposition in brain and liver, albeit initial gastric irritation in fewer patients (Munk et al., 2022). However, there are rare small molecule compounds in response to facilitating GSH synthesis and metallothionein induction in the field of Cu reservation and cuproptosis to date, such compounds are awaiting further exploration.

### 4.2 Small molecule compounds to inhibit cuproptosis by targeting mitochondrial respiration and TCA cycle

According to the outcome of Tsvetkov et al., the cells with active mitochondrial respiration and high levels of lipoylated TCA enzymes were more sensitive to the Cu-dependent cell death; treatment with fatty acids, mitochondrial function inhibitors and mitochondrial antioxidants showed different degrees of influence on Cu cytotoxicity; notably ETC inhibitors and mitochondrial pyruvate uptake inhibitors retarded the cell death effects by Cu-ionophores (Tsvetkov et al., 2022). Thereby the small molecule compounds that impair ETC and mitochondrial pyruvate uptake enable to inhibit cuproptosis by targeting mitochondrial respiration and TCA cycle. The insecticide rotenone is an ETC I inhibitor that drives cell apoptosis via the induction of ROS in mitochondria (Kishore Kumar et al., 2017). The antibiotic complex antimycin A can block mitochondrial respiration and reduce cellular ATP level through binding to ETC III, leading to elevated induction of superoxide and cell apoptosis (Lanju et al., 2014). Wang et al. demonstrated that the mitochondrial pyruvate carrier inhibitor UK5099 elevated glycolytic enzymes and promoted glycolysis and lactate production (Wang et al., 2018). Hence these ETC and mitochondrial pyruvate uptake inhibitors have the potential to reprogram the mitochondrial respiration and TCA cycle to glycolysis, which will induce the dynamic changes of cuproptosis-related genes and reduce the sensitivity to Cu stress.

### 4.3 Small molecule compounds to alleviate cuproptosis by targeting oxidative stress

In the study of Tsvetkov et al., although treatment with the oxidative stress inhibitor N-acetyl cysteine failed to abrogate the cell death mediated by elesclomol-Cu(II), excess ROS is one of the cell death effects generated by Cu Fenton reaction (Tsvetkov et al., 2022). In addition to chelators reducing intracellular Cu content, we are looking for some small molecule compounds to alleviate the cellular damages of cuproptosis by reducing the ROS derived from Cu Fenton reaction. Coumarin represents a large amount of 1,2-benzopyrone derivatives and studies have demonstrated that their antioxidant capacity to Cu Fenton reaction relies on chelation, apart from direct scavenging of ROS (Filipský et al., 2015). Deferiprone has high affinity to interact with Cu and is reported to reduce the production of hydroxyl free radicals in the presence of H_2_O_2_ (Timoshnikov et al., 2019). Moreover, Castañeda-Arriaga R et al. found that the phenolic acids including ferulic, protocatechuic and gallic acids present in nopal could inhibit the Cu(II) reduction, prevent hydroxyl free radicals formation by Cu chelation and react with hydroxyl free radicals, thus inhibiting the damages derived from Cu Fenton reaction (Castañeda-Arriaga et al., 2021). These compounds can be considered for the potential treatment to alleviate Cu overload and toxicity by targeting oxidative stress.

### 4.4 Transcription factors to inhibit cuproptosis

Based on the principle and action mode of cuproptosis, the transcription factors which can reduce the sensitivity to Cu toxicity, facilitate Cu efflux and reservation have attracted lots of interests, owing to their potential to inhibit cuproptosis. It has been confirmed that the glycolytic cells show lower sensitivity to Cu toxicity, so we may prevent Cu cell death effects by reprogramming the cellular metabolism to favor glycolysis, and HIF-1α is such a transcription factor to hamper cuproptosis sensitivity due to its hypoxic pathways modulating glycolytic enzymes in tumor cells (Huang et al., 2024). For Cu trafficking, the negative regulatory factor of cuproptosis MTF1 is capable of binding to the metal-responsive element of ATP7B promoter, their failed interaction may contribute to the unusual Cu efflux in Wilson disease (Stalke et al., 2020); for Cu reservation, MTF1 can bind to the metal-responsive element in the upstream of metallothionein and the elevation of metallothionein reduces the intracellular free Cu ions in response to Cu stress (Adams et al., 2015). These transcription factors herein are strong candidates in regulating Cu overload and homeostasis imbalance.

### 4.5 NcRNAs to inhibit cuproptosis

Similarly, most the ncRNAs we collect to modulate Cu membrane transporter genes and cuproptosis-related genes are involved in drug resistance not Cu-dependent cell death, and some of them are derived from bioinformatics analysis without experiment verification. We note that Xie M. et al. verified that FDX1, a cuproptosis master regulator, was downregulated in clear cell renal cell carcinoma to promote malignant biological properties; from interaction assays, FDX1 was identified as a target gene of miR-21-5p; miR-21-5p negatively regulated FDX1 expression to suppress Cu cell death effects for better tumor progress (Xie et al., 2022). So delivery of miRNA inhibitors reversing the ncRNA target effects on cuproptosis positive regulator devises novel therapeutic approach to carcinoma through the induction of cuproptosis, but it remains a big challenge.

## 5 Discussion

Cu homeostasis dysfunction and cuproptosis are closely correlated with the occurrence of some genetic, neurodegenerative, cardiovascular, tumoral diseases, and the medical options have been developed into recovering Cu homeostasis or triggering Cu dysfunction (Chen et al., 2022). For instances, in Wilson disease and Menkes disease, the pathogenic variants of ATP7A/7B disturb the intracellular Cu content and homeostasis, the treatment is to recover normal intracellular Cu content (Wilson, 1934; Danks et al., 1972); in tumoral diseases, mounting evidences suggest that Cu dyshomeostasis plays a prominent role in energy metabolism and angiogenesis, the treatment targets oxidative stress, mitochondrial respiration, UPS and angiogenesis using Cu complexes (Ge et al., 2022). Although the main morphological features of cuproptosis are similar to apoptosis, including cytomembrane/chromatin rupture, endoplasmic reticulum damage and mitochondrial contraction, but its mechanism is varied from other known cell death pathways (Liao et al., 2019; Zhao et al., 2020). Excess Cu ions induce cuproptosis by promoting the insoluble oligomerization of lipoylated proteins in TCA cycle, suppressing Fe-S cluster protein, causing proteotoxic stress and elevating HSP70, ROS (Figure 3) (Tsvetkov et al., 2022). Hence, the discovery of cuproptosis will widen the path for future drug development targeting Cu homeostasis, intensive research of the potential role of cuproptosis in diverse diseases and the molecular mechanism will contribute to exploring novel therapeutic targets and thereby driving drug innovation. Nowadays, the cuproptosis inducers and inhibitors have been continuously improved and employed for scientific studies and medical options, and in this study they are collected and categorized into small molecule compounds, transcription factors and ncRNAs, in terms of the key targets in cuproptosis metabolic pathways.

The discovery of cuproptosis by Tsvetkov P and colleagues emphasizes the regulatory mechanism of positive regulatory factors FDX1, lipoylated proteins (LIPT1, LIAS, DLD), lipoylated targets (DLAT, PDHA1, PDHB), negative regulatory factors GLS, CDKN2A, MTF1 and mitochondrial metabolism (lipoylated pathway, TCA cycle) in Cu-induced cell death (Tsvetkov et al., 2022). The cells using TCA cycle and OXPHOS as their energy production mode exhibit higher sensitivity to Cu stress than the cells relied on glycolysis. In this process, FDX1 plays a critical role in reducing Cu(II) to Cu(I), lipoylating DLAT and destabilizing Fe-S cluster protein to trigger cuproptosis. We pay more attention to small molecule compounds which influence the progress of Cu-induced cell death, rather than transcription factors, ncRNAs or other types of regulators, since they are more readily screened and dosed to produce therapeutic outcome than other types of candidates. For inducers of cuproptosis, to raise intracellular Cu content, we collect some small molecule compounds targeting Cu ionophores: elesclomol, disulfiram, clioquinol, 8-quinolinol, schiff base, pyrithione, zinc metallochaperone, Cu(II)ATSM/Cu(II)GTSM, UM4118, they are capable of delivering Cu ions into cells; Cu membrane transporters: epigallocatechol gallate, β-elemene, CID 2011756, omeprazole, 6-hydroxydopamine hydrobromide, they can upregulate SLC31A1 expression or downregulate ATP7A/7B expression to drive Cu deposition; Cu reservation: 6-hydroxydopamine hydrobromide, actinomycin D, PAPA NONOate, DL-buthionine-(S,R)-sulfoximine, they enable to aggravate the release of intracellular Cu ions; Cu chaperones: DC_AC50, it has the ability to reduce Cu trafficking by inhibiting ATOX1 and CCS. To enhance mitochondrial respiration and TCA cycle, the small molecule compounds phloretin, PX-478, 2-deoxy-D-glucose and galactose can be employed as glycolysis inhibitors to shift the energy metabolism to TCA cycle and OXPHOS. The transcription factors ELF3, Sp1, ATF3/SPI1, facilitating the expression of SLC31A1 and the ncRNAs LINC02362/miR-18a-5p, facilitating the expression of FDX1 are accompanied to enhance the sensitivity to Cu stress beyond small molecule compounds (Figure 4; Table 1). For inhibitors of cuproptosis, to reduce intracellular Cu content, we collect some small molecule compounds targeting Cu chelators: GSH, tetrathiomolybdate, bathocuproinedisulfonic acid, trientine, penicillamine, 2,6-Pyridinedicarboxylic acid, 2,6-bis[2-[(4-carboxyphenyl) methylene] hydrazide], 3,5-di-tert-butylsalicylaldehyde rhodamine hydrazone, captopril, LCC-12, curcumin, they are capable of chelating intracellular Cu ions; Cu reservation: Zinc acetate, it can elevate metallothionein level in intestinal cells. To retard mitochondrial respiration and TCA cycle, the small molecule compounds rotenone, antimycin A, UK5099 can be employed as mitochondrial respiration inhibitors to shift the energy metabolism to glycolysis. To recover oxidative homeostasis, the small molecule compounds coumarin, deferiprone, phenolic acids are helpful to eliminate the ROS derived from Cu Fenton reaction. The transcription factors HIF-1α, facilitating hypoxia, MTF1, facilitating ATP7B and metallothionein expression and the ncRNA miR-21-5p, decreasing FDX1 expression are accompanied to reduce the sensitivity to Cu stress beyond small molecule compounds (Figure 5; Table 2). The strategies to collect cuproptosis inducers originate from raising the intracellular Cu content and the intensity of mitochondrial respiration and TCA cycle, while the strategies to collect cuproptosis inhibitors originate from reducing intracellular Cu content, the intensity of mitochondrial respiration, TCA cycle, oxidative stress and UPS inhibition. Importantly, the medical values of cuproptosis inducers and inhibitors are mainly concentrated in the treatment of genetic, neurodegenerative, cardiovascular and tumoral diseases, achieving by recovering normal Cu homeostasis, inducing Cu overload or deficiency. But to date, we have not collected applicable small molecule compounds for the cuproptosis inhibitors that target Cu membrane transporters, Cu chaperones or UPS inhibition during Cu stress and they require further exploration.

It can be concluded from the studies that cuproptosis inducers and inhibitors have the potential to modulate Cu cell death effects relying on their composition and property, but the action mode may vary from distinct drug candidates and cell types. Some compounds we collect have been applied for the clinical trials of Wilson disease such as tetrathiomolybdate (NCT00004339) (Brewer et al., 2006), trientine (NCT01472874) (Ala et al., 2015), penicillamine (Schilsky et al., 2022), Zinc acetate (NCT00212355) (Shimizu et al., 2010) and reduced Cu accumulation in liver and brain through complex and metallothionein chelation, respectively. Cu overload and deficiency constitute an exploitable dependency in cancer therapy, and some Cu ionophores we collected have been pursued in cancer clinical trials and produced considerable therapeutic outcome such as elesclomol (NCT00084214, NCT00888615) (O’Day et al., 2009; Monk et al., 2018) and disulfiram (NCT01907165) (Huang et al., 2016). Hence, cuproptosis has attracted dramatic interests in providing a new avenue for medical options, and promising compounds that can modulate Cu-induced cell death in preclinical studies have been also collected and displayed in our study. We should dedicate to improve the combination strategies so that the cuproptosis inducers and inhibitors we collect can provide better therapeutic approaches. Firstly, Cu ionophore plus chemotherapy. Clinical trails had revealed encouraging efficacy for elesclomol plus paclitaxel in metastatic melanoma versus paclitaxel alone, and elesclomol had stronger anti-tumor activity in patients with lower LDH level, indicating that elesclomol was more sensitive to the cells harboring intensive mitochondrial respiration and lipoylated TCA enzymes (O’Day et al., 2009; O’Day et al., 2013). Secondly, Cu ionophore plus ferroptosis inducer. The data of Wang W et al. provided the evidence that ferroptosis inducers sorafenib and erastin reduced FDX1 degradation and GSH synthesis to enhance elesclomol-Cu-induced cell death in liver cancer cells, suggesting a link between cuproptosis and ferroptosis in the therapy depending on metal element dysfunction (Wang W. et al., 2023). Thirdly, Cu complexes have great potential use for disease treatment, however, it is important to note that long-term use of Cu complexes can disrupt the base metal homeostasis and cause serious side effects in patients undergoing treatment. This field is in its early stage of development and lack of specificity is a major challenge. The scientific communities have developed innovative nanomedicine delivery vectors to improve the specificity of Cu complexes to morbid cells and obtain more accurate therapeutic outcome. Cui L and colleagues formulated a mitochondria-targeted Cu depleting nanoparticle consisting of Cu chelator, semiconducting polymers and phospholipid-polyethylene glycol, the positive surface charge favored its accumulation in mitochondria and herein reduced the Cu content, which dampened OXPHOS activity and drived oxidative stress to inhibit triple-negative breast cancer cells with minimal side effects (Cui et al., 2021). Therefore, nanocarriers selectively delivering cuproptosis inducers and inhibitors to the lesions can elevate precise therapeutic outcome and reduce toxicity for medical applications based on Cu cell death effects, but the composition, property, cell type of these delivery system, and further clinical trials still necessitate in-depth exploration.

In addition to the small molecule compounds, transcription factors and ncRNAs we collect in this study, we note that other types of cuproptosis-related molecules can also modulate Cu cell death effects. Proteases are such a kind of potential drug target. For instances, the phosphodiesterase 3B has been confirmed to increase the sensitivity of Cu ionophore to bladder cancer cells (Feng et al., 2024); the methyltransferase METTL16 is reported to promote FDX1 mRNA stability via m^6^A modification and FDX1 accumulation to enhance the sensitivity of elesclomol treatment in gastric cancer (Sun et al., 2023). Transcription factors, ncRNAs and proteases have been taken into therapeutic consideration for cuproptosis, however, it remains a big challenge to successfully deliver gene expression vectors or ncRNAs in human body. Chiefly, small molecule compounds are strong candidates in regulating Cu-induced cell death owing to the suitable screen and administration routes. The candidate compounds can be excavated from rounds of verification: bioinformatic analysis, molecular simulation and docking, interaction analysis. In this study, we have collected kinds of cuproptosis inducers and inhibitors based on the key targets and metabolic pathways of Cu-induced cell death, but the combination strategy and drug carrier remain incompletely documented, which may thus be promising tools for curing patients with Cu homeostasis dysfunction. Moreover, in view of the significance of cuproptosis and ferroptosis, the collapse and recovery of other metal ion homeostasis also have definite research value for the exploration of cell death identification, disease mechanisms and therapeutic targets. Jiang JK et al. found that excess manganese ions downregulated the expression of sirtuin1 to induce apoptosis of nerve cells, suggesting that blocking manganese ions-mediated cell death might have a certain prospect in developing therapeutics of neurodegenerative diseases (Zhao et al., 2019). Xu H et al. reported a lysosomal calcium/zinc channel-mediated and zinc-dependent cell death in metastatic melanoma so that targeting zinc dysfunction showed potential value for melanoma therapy (Du et al., 2021). Another metal element calcium signaling activates specific proteins to regulate many physiological processes, and its abnormal transduction is expected to provide a new approach for tumor treatment (Bai et al., 2022). Herein, we will keep our eye on Cu- and other metal elements-mediated cell death, particularly in the field of inducers and inhibitors, which are valuable for identifying cell death pathways, exploring disease mechanisms and therapeutic targets based on metal element dysfunction.

## 6 Conclusion

In summary, cuproptosis is a newly discovered pathway of Cu-induced cell death which provides an innovative avenue for medical options. We mainly collect the small molecule compounds that induce or inhibit cuproptosis based on the key targets and metabolic pathways of Cu stress in this study, accompanied by relevant transcription factors and ncRNAs. They have the promising potential to be employed as inducers or inhibitors in the scientific studies and medical options of Cu homeostasis dysfunction. However, the action mode of Cu-induced cell death still requires in-depth investigation to understand the hallmark of drug targets and metabolic pathways. Meanwhile the drug species, combination and delivery strategies of cuproptosis inducers or inhibitors also await further exploration.
